# Surgery induces neurocognitive disorder via neuroinflammation and glymphatic dysfunction in middle-aged mice with brain lymphatic drainage impairment

**DOI:** 10.3389/fnins.2024.1426718

**Published:** 2024-06-20

**Authors:** Xiaoqiu Zhu, Jingrun Lin, Pengfeng Yang, Shaotao Wu, Huijun Lin, Wen He, Daowei Lin, Minghui Cao

**Affiliations:** ^1^Department of Anesthesiology, Sun Yat-sen Memorial Hospital, Sun Yat-sen University, Guangzhou, Guangdong, China; ^2^Department of Ultrasound Medicine, Guangdong Provincial Key Laboratory of Major Obstetric Diseases, Guangdong Provincial Clinical Research Center for Obstetrics and Gynecology, The Third Affiliated Hospital of Guangzhou Medical University, Guangzhou, Guangdong, China

**Keywords:** postoperative neurocognitive disorder, neuroinflammation, brain lymphatic drainage system, glymphatic system, meningeal lymphatic vessels, HMGB1/TLR-4/NF-κB pathway

## Abstract

**Background:**

Brain lymphatic drainage impairment is a prevalent characteristic in both aging and neurodegeneration. Surgery is more likely to induce excessive neuroinflammation and postoperative neurocognitive disorder (PND) among patients with aging and neurodegeneration. We hypothesized that surgical trauma may aggravate PND through preexisting cerebral lymphatic drainage impairment. However, there remains limited understanding about the role of surgery in changes of neurocognitive function in the populations with preoperative brain lymphatic drainage impairment. This study aims to expand our insight into surgery-induced glymphatic dysfunction, neuroinflammation and PND in middle-aged mice with preoperative brain lymphatic drainage impairment.

**Materials and methods:**

Deep cervical lymph nodes ligation (LdcLNs) was performed on middle-aged mice to establish preoperative brain lymphatic drainage impairment. A month later, laparotomy was performed on these mice with or without LdcLNs followed by analysis of brain neuroinflammation, glymphatic function, neuronal damage, and behavioral test.

**Results:**

LdcLNs disrupted meningeal lymphatic drainage. In middle-aged mice with LdcLNs, surgery exacerbated more serious glymphatic dysfunction accompanied by aggravation of A1 astrocytes activation and AQP4 depolarization. Furthermore, surgery caused neuronal damage via reducing expression of neuronal nuclei (NeuN), post-synaptic density protein 95 (PSD95) and synaptophysin (SYP), as well as impairment in exploratory behavior and spatial working memory in middle-aged mice with LdcLNs. Additionally, surgery induced neuroinflammation with elevated microglia activation and increased the levels of tumor necrosis factor (TNF)-α, interleukin (IL)-1β and IL-6, as well as activated more expression of HMGB1/TLR-4/NF-κB pathway in middle-aged mice with LdcLNs.

**Conclusion:**

Surgery exacerbates neuroinflammation and glymphatic dysfunction, ultimately resulting in neuronal damage and neurocognitive disorder in middle-aged mice with preoperative brain lymphatic drainage impairment. These results suggest that brain lymphatic drainage impairment may be a deteriorating factor in the progression of PND, and restoring its function may serve as a potential strategy against PND.

## Introduction

Aging and neurodegenerative related diseases, such as Alzheimer’s disease (AD) and some cerebrovascular diseases, exhibit increased vulnerability to postoperative neurocognitive disorders (PND), which affects millions of surgical patients every year (Silbert et al., 2015; Li et al., 2021; Sun et al., 2021; Roberts et al., 2024). PND is characterized by a surgery-induced deterioration in cognitive function, including memory impairment, attention deficits, and other cognitive domain impairments. Furthermore, individuals with PND exhibit a higher risk for developing dementia (Evered et al., 2018; Deiner et al., 2021; He et al., 2024). PND has been associated with increased morbidity and mortality rates, prolonged hospital stays, and diminished quality of life (Zhong et al., 2022; Zeng et al., 2023). In the foreseeable future, surgical treatment will continue to be the primary radical approach for various surgical conditions; hence, it is imperative to enhance postoperative cognitive decline management for the populations with aging, neurodegenerative diseases and cerebrovascular diseases.

Over the past decade, numerous studies demonstrated that the impairment of the brain lymphatic drainage system not only implicated in the pathogenesis of aging and neurodegenerative diseases, but also involves in cerebrovascular and neuroinflammatory diseases (Kress et al., 2014; Sun et al., 2018; Hussain et al., 2023; Jiang et al., 2023). The brain lymphatic drainage system mainly composed of the glymphatic system and meningeal lymphatic vessels (mLVs), which can drain cerebrospinal fluid (CSF) and intercellular interstitial fluid from the brain-wide parenchyma to deep cervical lymph nodes (dcLNs). This system plays a crucial role in maintaining homeostasis of the central nervous system (CNS), such as clearance of metabolic waste and toxic substances, solute transport, and immune surveillance (Da Mesquita et al., 2018; Sun et al., 2018; Yankova et al., 2021).

It has been demonstrated that brain lymphatic drainage impairment exacerbates the deposition of amyloid-β, tau, and synuclein in the corresponding disease models (Da Mesquita et al., 2018; Wang et al., 2019; Zou et al., 2019), as well as brain edema and infarct volume in the stroke model (Si et al., 2006), ultimately leading to corresponding behavioral impairments. Surgical trauma can also increase the release of damage-associated molecular patterns (DAMPs) and induce the upregulation of inflammatory factors, oxidative stress products, as well as HMGB1 and other DAMPs into the CNS (Terrando et al., 2016; Jia et al., 2023; Liu et al., 2023). These aforementioned substances may also exhibit excessive accumulation in condition of preexisting impairment in brain lymphatic drainage, thereby inducing neuronal damage and PND. However, there remains limited understanding about the role of surgery in changes of neurocognitive function for the populations with preoperative brain lymphatic drainage impairment.

Therefore, this study aims to expand our insight of surgery-induced glymphatic dysfunction, neuroinflammation, and PND in middle-aged mice with preoperative brain lymphatic drainage impairment. We employed the technique of dcLNs ligation (LdcLNs) to induce brain lymphatic drainage impairment since the dcLNs play a crucial role in draining CSF from the brain parenchyma into the peripheral lymphatic system (Wang et al., 2019; Zou et al., 2019). In this study, we demonstrated that surgery activated the HMGB1/TLR-4/NF-κB pathway, induced neuroinflammation with microglia activation and release of inflammatory factors, exacerbated glymphatic dysfunction with A1 astrocyte activation and AQP4 depolarization, as well as caused neuronal damage and PND in middle-aged mice with preoperative brain lymphatic drainage impairment.

## Materials and methods

### Animals

C57BL/6J male mice (aged 9–10 months) were provided by Sun Yat-sen University, and were housed under standard conditions (room temperature 20–24°C, humidity 30–50%, 12 h light/12 h dark cycle). The animals were randomly divided into four groups: Con group (not conducted exploratory laparotomy and ligation of the deep cervical lymph nodes); Surgery group (only conducted exploratory laparotomy but not ligation of the deep cervical lymph nodes); Con + LdcLN group (conducted ligation of the deep cervical lymph nodes but not exploratory laparotomy a month later); Surgery + LdcLN group (conducted ligation of the deep cervical lymph nodes and exploratory laparotomy a month later) The experiment was approved by the Animal Ethical and Welfare Committee of Sun Yat-sen University and conducted in accordance with the National Institutes of Health animal care guidelines.

### Ligation of the deep cervical lymph nodes (LdcLNs)

According to previous literature (Zou et al., 2019), the procedure of LdcLNs was performed to establish preoperative brain lymphatic drainage impairment. After being anesthetized with 1–2% isoflurane, mice were fixed in a supine position. A midline incision (approximately 1 cm) was made along of the neck after cleaned with iodine and 75% ethanol. Then, the fat and soft tissue were bluntly separated under a microscope, and the both sides of dcLNs were exposed after retraction of the sternocleidomastoid muscles. Finally, the afferent vessels of dcLNs in both sides were carefully ligated by 8–0 nylon suture. The incision was sutured and disinfected with iodine and 75% ethanol. The Con group and Surgery group were only exposed the dcLNs without ligation. The animals were returned to their cages after emergence from anesthesia and were fed for 1 month followed by exploratory laparotomy.

### Exploratory laparotomy

Mice in the Surgery group and Surgery + LdcLN group underwent exploratory laparotomy. Mice were induced anesthesia using 2% isoflurane (RWD, China) in a sealed chamber for 3 min and were performed the surgical procedure under 1.2–1.5% isoflurane with 0.5 L⋅min^–1^ O_2_, their temperature was maintained by a warm pad. A midline abdominal incision of approximately 2 cm was made to expose the abdominal cavity, and then the liver, stomach, spleen, bowel, kidney and bladder were explored. Finally, a section of small intestine (approximately 5 cm) covered with moist gauze was exteriorized and gently rubbed for 10 min. The muscle, fascia, and skin were sutured using 5–0 nylon sutures. All animals received a postoperative subcutaneous injection (3 mg/kg bupivacaine) along the incision for analgesia. The animals were returned to their cages after emergence from anesthesia. Mice in the Con group and Con + LdcLN group did not undergo exploratory laparotomy.

### Confirmation of brain lymphatic drainage blockade by dcLN ligation using intracisternal infusion of Evans blue (EB) dye

The blockade of brain lymphatic drainage by dcLN ligation was confirmed through injection of 2% EB (Sigma, USA) dye into the cisterna magna. Specifically, ligation was performed on the right dcLN while the left side remained non-ligated. After 60 min, 10 μL EB dye was injected into the cisterna magna at a rate of 1 μL/min through a Hamilton syringe coupled to a 30-gauge needle and connected to a constant current infusion pump (RWD, China). The needle was withdrawn 10 min after the end of infusion. The accumulation of EB dye in the both dcLNs was observed under the stereomicroscope at 60 min post-injection.

### Novel object recognition (NOR) test

Mice were handled 10 min by one particular experimenter for 3 days before the behavioral tests. Mice were placed in a quiet room for 1-h adaptation before test. On day 1, each mouse was allowed to explore the chamber (40 cm × 40 cm × 40 cm) for a 10-min habituation. On day 2, each mouse was allowed to explore two similar objects in chamber for a 10-min training. On day 3, one of the objects was replaced by a novel one after 24 h, and each mouse was allowed to explore two different objects in chamber for a 10-min test. Each mouse’s movements were traced and recorded by the TopScanTM2.0 software (PhenoScan, USA). Recognition index = The time of novel object exploration / the total exploration time.

### Y-maze test

Y maze was performed on the day following the NOR test. The spontaneous alternation test of the Y-maze was performed to evaluate working memory. The Y-maze consisted of three enclosed arms (30 cm × 8 cm × 12 cm) forming a 120° angle. Each mouse underwent a 1-h adaptation period in a quiet room. Then, each mouse was gently placed in the center and allowed to explore three arms during a 10-min test period, and its sequence and number of arm entries were recorded by an observer blinded to the experiment. An entry was defined as when all 4 paws of the mouse entered an arm. The spontaneous alternation was defined as successive entry into the three arms (Figure 5E). The percentage of spontaneous alternation was calculated as follows: spontaneous alternation (%) = ([number of spontaneous alternations] / [total arm entries − 2]) × 100. The device was wiped with 75% ethanol to keep clean between uses.

### Intracisternal infusion of fluorescent tracer

Mice were placed on a heating pad and their eyes were lubricated with vaseline after anesthetized by sodium pentobarbital (100 mg/kg). The head of mouse was fixed in a stereotaxic instrument. The hair of the head and back neck was shaved and the skin were sterilized with iodine and 75% ethanol. A 1 cm incision was made along the midline of the head and posterior neck, followed by exposure of the membrane overlying the cisterna magna through retraction of the muscle layers. Ten μL Ovalbumin-Alexa Fluor 647 (Thermo Scientific, Waltham, MA, USA; 0.5% in artificial cerebrospinal fluid) was injected into the cisterna magna at a rate of 1 μL/min through a Hamilton syringe coupled to a 30-gauge needle and connected to a constant current infusion pump (RWD, China). The needle was withdrawn 10 min after the end of infusion. The mice were perfused 1 h after intracisternal infusion. dcLNs were sliced into 10 μm sections, and the brains were sliced into 100 μm coronal sections for fluorescent tracer assessment by a blinded investigator using fluorescence microscope and ImageJ software.

### Hippocampal infusion of fluorescent tracer

Mice were placed on a heating pad and their eyes were lubricated with vaseline after being anesthetized by sodium pentobarbital (100 mg/kg). The head of mouse was fixed in a stereotaxic instrument. The hair on the head was shaved and the skin were sterilized with iodine and 75% ethanol. A 1 cm incision was made along the midline of the head to expose the dorsal skull. 1.5 μL Ovalbumin-Alexa Fluor 647 was infused into the hippocampus (coordinates in mm from Bregma: ML −1.5; AP −2; DV −2.0) at a rate of 0.1 μL/min through a Hamilton syringe coupled to a 30-gauge needle and connected to a constant current infusion pump. The needle was withdrawn 10 min after the end of infusion. The mice were perfused 3 h after hippocampal injection. The brains were sliced into 100 μm coronal sections for fluorescent tracer assessment by a blinded investigator using fluorescence microscope and ImageJ software.

### Preparation of the dorsal dural meninges

The cranium was removed with surgical scissors and fixed overnight at 4°C in 4% paraformaldehyde solution. Subsequently, dorsal dural meninges were carefully dissected from the skull with delicate forceps under a dissecting microscope. Finally, whole-mount meninges were immerged into Phosphate-Buffered Saline (PBS) for further immunofluorescence staining.

### Immunofluorescent staining

The dorsal dural meninges and the slices of brains and dcLNs were fixed in 4% paraformaldehyde overnight at 4°C and then incubated in 30% sucrose for 2–3 days at 4°C. After frozen in optimal cutting temperature (OCT) compound, 30 μm coronal brain sections were cut in the CryoStar NX50 (Thermo NX50). The brain slices were washed 3 times with PBS, and were incubated with a blocking solution (5% bovine serum albumin [BSA] + 5% donkey serum in PBS + 0.3% Triton-X100) for 1 h at room temperature. Next, the slices were incubated with the primary antibodies overnight at 4°C. slices were washed with PBS for 3 times, and incubated with the secondary antibody for 1 h and Cell nuclei were stained by 4′,6-diamidino-2-phenylindole (DAPI, 1:1000, Sigma, USA) for 30 min at room temperature in the dark condition. Images were acquired with a fluorescence microscope (Leica DM6B, Germany). All quantitative analyses were performed in a blinded manner using ImageJ software. The primary antibodies for immunofluorescence staining included rabbit anti-IBA1 antibody (1: 1000, Wako, 019–19741), rabbit-anti-AQP4 antibody (1:500, Oasis Biofarm, OB-PRB058), guinea pig-anti-NeuN antibody (1:500, Oasis Biofarm, OB-PGP006), guinea pig-anti-NeuN antibody (1:500, Oasis Biofarm, OB-PGP006), guinea pig-anti-GFAP antibody (1:500, Oasis Biofarm, OB-PGP055), mouse-anti-C3 antibody (1:500, Hycult Biotech, HM1045), rat-anti-LVYE1 antibody (1:200, eBioscience, 14-0443-82).

Astrocytic AQP4 polarization was quantified using ImageJ analysis as described in previous literature (Wang et al., 2012). Briefly, after color channels were split, the AQP4 channel was thresholded at two levels: low stringency (included all AQP4-immunoreactive pixels) and High stringency (captured all pixels representing perivascular end feet). The AQP4 polarization was derived as the ratio between low stringency and high stringency. Furthermore, polarization index was applied for more accurate analysis of the distribution of perivascular AQP4 centered on a specific vasculature in each group. The line-plot tool of ImageJ was used to analyze 50 μm segments centered on vasculatures identified by vascular-shaped localization of AQP4. The chosen vasculatures had an approximate width of 6 μm (ranging from 5 to 8 μm) and a length of 32 μm (ranging from 20 to 55 μm) and 3 matched vasculatures were chosen per image. The polarization index is calculated by subtracting the 10 μm baseline from the peak fluorescence. The baseline was defined as the average fluorescence intensity over 10 μm, −20 μm to −10 μm from the peak vascular end foot fluorescence. All values were normalized to the highest signal for better visualization (Hablitz et al., 2020).

### Western blotting

Brain tissues were lysed with an appropriate amount of RIPA lysis buffer (Beyotime, P0013B) with protease and phosphatase inhibitors on ice with ultrasonication and centrifuged at 12,000 rpm for 15 min at 4°C. The supernatants were collected and added with 5 × SDS-PAGE loading buffer. Protein concentrations were measured using the BCA assay (Beyotime, P0010). Protein samples (approximately 20 μg) were separated by 10 or 12% SDS-PAGE electrophoresis, transferred onto PVDF membranes, and blocked with a 5% non-fat milk solution. They were then incubated overnight at 4°C with specific primary antibodies, followed by a 1-h incubation at room temperature with specific secondary antibodies. The signals were detected by Odyssey Infrared Imaging System (LI-COR Biosciences, Lincoln, NE, USA) and analyzed by ImageJ software. The primary antibodies for western blotting included rabbit anti-PSD95 antibody (1:1000, Cell Signaling Technology, 3450), rabbit-anti-Synaptophysin (SYP) antibody (1:5000, Proteintech, 17785-1-AP), rabbit-anti-HMGB1 antibody (1:1000, Cell Signaling Technology, 6893), rabbit-anti-TLR4 antibody (1:2000, Proteintech, 19811-1-AP), rabbit-anti-phospho-NF-κB antibody (1:1000, Cell Signaling Technology, 3033), mouse-anti-GAPDH antibody (1:20000, Proteintech, 60004-1-Ig), mouse-anti-α-Tubulin antibody (1:20000, Proteintech, 66031-1-Ig).

### Enzyme linked immunosorbent assay (ELISA)

The assay procedure was carried out according to the manufacturer’s instructions. Mouse Enzyme-Linked ImmunoSorbent Assay Kits (PI301, PI326, PT512; Beyotime) were, respectively, applied to measure the concentrations of Interleukin-1β (IL-1β), IL-6 and tumor necrosis factor-α (TNF-α) in hippocampus. The standard or samples were added to the 96-well plate and incubated with the antibody. Subsequently, biotinylated anti-target protein antibody was added to bind to the target protein to form a sandwich immune complex after three-time washing. HRP-Streptavidin was subsequently added to catalyze and the 3,3′5,5′-tetramethylbenzidine (TMB) substrate was added to generate blue colorimetric reaction which became yellow after the addition of the termination solution. Quantitative detection was achieved by measuring the absorbance value at 450 nm with a microplate reader.

### Brain water content measurement

The brains of anesthetized mice were removed and weighed, followed by heated in an oven at 70°C for 72 h and reweighed. Percentage water content = [(wet weight–dry weight)/wet weight × 100%].

### Statistics and data presentation

All data in normal distribution were presented as mean ± standard error of the mean (SEM) and analyzed using GraphPad Prism 5. Differences between groups were analyzed one-way analysis of variance (ANOVA) followed by the Dunnett’s test or Tukey’s multiple comparison test. *Post-hoc* tests were used only if F achieved *p* < 0.05 and there was no significant inhomogeneity of variance. Statistical significance was considered at *p* < 0.05.

## Results

### Ligation of dcLNs disrupts meningeal lymphatic drainage

**FIGURE 1:**
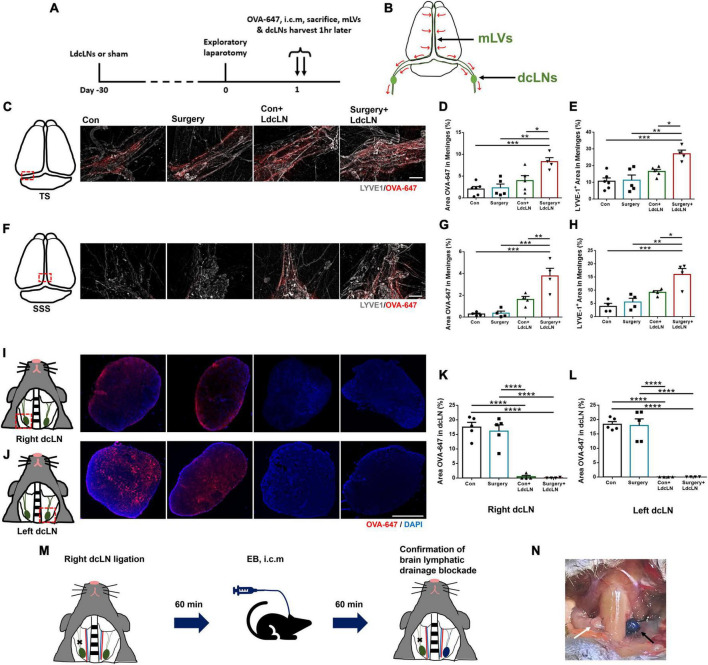
Ligation of dcLNs disrupts meningeal lymphatic drainage dysfunction in middle-aged mice. **(A)** The timeline of brain lymphatic drainage test and tissue collection on day 1 after surgery. **(B)** Schematic diagram of brain lymphatic drainage from cerebral parenchyma to deep cervical lymph nodes. **(C–H)** Representative images of OVA-647 and LYVE1 fluorescence area in the transverse sinus (TS) and superior sagittal sinus (SSS) of meninges, and quantitative results of OVA-647 and LYVE1 fluorescence area in the meninges. Scale bar = 400 μm **(I–L)** Representative images of OVA-647 fluorescence area in dcLNs and quantitative results of OVA-647 fluorescence area in right and left dcLNs. Scale bar = 400 μm **(M)** Timeline for confirmation of brain lymphatic drainage blockade by LdcLN: the right dcLN was ligated while the left side remained no-ligated. After 60 min, 4% Evans blue (EB) dye was injected into the cisterna magna and the EB accumulation in both dcLNs was observed at 60 min post-injection. **(N)** The EB accumulation was observed apparently in the no-ligated dcLN (left, black arrow), while minimal accumulation of EB dye was detected in the ligated dcLN (right, white arrow) after cisterna magna injection (i.c.m). *N* = 4–6, data were analyzed with one-way ANOVA, and expressed as means ± SEM. *p < 0.05, **p < 0.01, ***p < 0.001, and ****p < 0.0001. Meningeal lymphatic vessels, mLVs; Deep cervical lymph nodes, dcLNs.

To determine whether LdcLNs causes impairment in brain lymphatic drainage, we observed the accumulation of OVA-647 in mLVs and dcLNs of four groups at 1 h after cisterna magna injection (Figures 1A, B). We observed that more OVA-647 was accumulated in transverse sinus (TS) and superior sagittal sinus (SSS) of mice with LdcLNs compared to those without LdcLNs, in both control and surgery groups. As for mLVs in both TS and SSS, significantly larger fluorescence areas of LYVE1^+^ staining was observed in the Surgery+LdcLNs group compared to the other three groups, and the Con+LdcLNs group also exhibited a tendency for increased fluorescence area when compared to the Con and Surgery groups (Figures 1C–H). Consistent with previous studies (Wang et al., 2019), the signal of OVA-647 was nearly undetectable in right and left dcLNs of mice with LdcLNs, while the signal was clearly observed in both dcLNs of mice without LdcLNs (Figures 1I–L). These results indicate that ligation of dcLNs for one month can disrupt meningeal lymphatic drainage the blockage of brain lymphatic drainage by LdcLNs and potentially causes compensatory proliferation of meningeal lymphatic vessels in mice.

Additionally, we observed difference in EB dye drainage to dcLNs *in vivo* following cisterna magna injection after ligating unilateral dcLNs. The EB accumulation was observed apparently in the no-ligated dcLN, while minimal accumulation of EB dye was detected in the ligated dcLN after cisterna magna injection, confirming that blockade of dcLNs could acutely block brain lymphatic drainage blockade in middle-aged mice (Figures 1M, N).

### Surgery exacerbates glymphatic dysfunction in middle-aged mice with preoperative brain lymphatic drainage impairment

**FIGURE 2:**
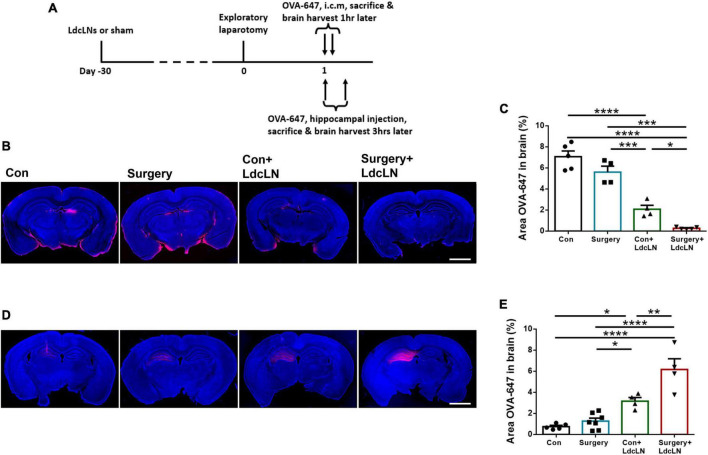
Surgery exacerbates glymphatic dysfunction in middle-aged mice with preoperative brain lymphatic drainage impairment. **(A)** The timeline of glymphatic function test and tissue collection after surgery. **(B)** Representative images of OVA-647 fluorescence area in cerebral parenchyma at 1 h after cisterna magna injection on day 1 after surgery. Scale bar = 2 mm **(C)** Quantitative results of OVA-647 fluorescence area in the cerebral parenchyma at 1 h after cisterna magna injection. **(D)** Representative images of OVA-647 fluorescence area in the hippocampus at 3 h after hippocampal injection on day 1 after surgery. Scale bar = 2 mm **(E)** Quantitative results of OVA-647 fluorescence area in the hippocampus at 3 h after hippocampal injection. *N* = 4–7, data were analyzed with one-way ANOVA, and expressed as means ± SEM. *p < 0.05, **p < 0.01, ***p < 0.001, and ****p < 0.0001.

Subsequently, we also assessed the effect of surgery on glymphatic function in mice with preoperative brain lymphatic drainage impairment. We evaluated glymphatic function by assessing the influx of OVA-647 into the brain parenchyma following cisterna magna injection and the efflux of OVA-647 from brain parenchyma after hippocampal injection in mice (Figure 2A). We observed that impairment of brain lymphatic drainage through LdcLNs resulted in a reduction in the influx of intracisternal OVA-647 into the brain parenchyma, which was further exacerbated by surgery in middle-aged mice with LdcLNs (Figures 2B, C). Furthermore, there was a decrease in glymphatic efflux dysfunction in the hippocampus of both LdcLNs groups. Surgery worsened the reduction in both the influx of OVA-647 into the brain parenchyma following cisterna magna injection and the efflux of OVA-647 from brain parenchyma after hippocampal injection in mice with LdcLNs (Figures 2D, E). These data revealed a synergistic effect of surgery and LdcLNs on impairment of glymphatic function, indicated that surgery exacerbates glymphatic dysfunction in middle-aged mice with preoperative brain lymphatic drainage impairment.

### Surgery aggravates activation of A1 astrocytes and AQP4 depolarization in middle-aged mice with preoperative brain lymphatic drainage impairment

**FIGURE 3:**
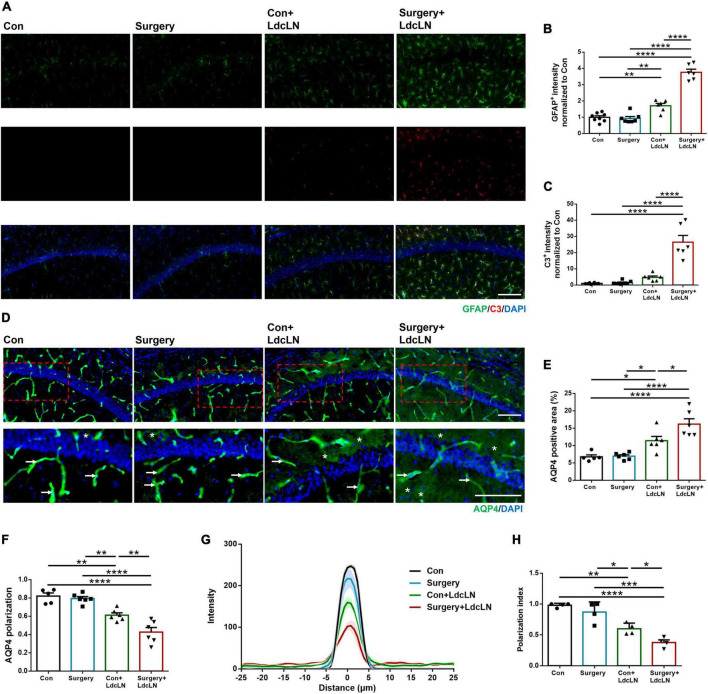
Surgery aggravates activation of A1 astrocytes and AQP4 depolarization in hippocampus of middle-aged mice with preoperative brain lymphatic drainage impairment. **(A)** Representative images of GFAP and C3 immunostaining in the hippocampus on day 1 after surgery. Scale bar = 100 μm **(B,C)** Quantitative results of GFAP and C3 fluorescence intensity in the hippocampus on day 1 after surgery. **(D)** Representative images of AQP4 immunostaining in the hippocampus on day 1 after surgery. The arrowheads indicate AQP4 immunoreactive signals expressed at astrocyte endfeet along with vessels for AQP4 polarization, and the stars indicate AQP4 immunoreactive signals expressed in areas of non-astrocyte endfeet for AQP4 depolarization. Scale bar = 100 μm **(E,F)** Quantitative results of AQP4 positive area and polarization in the hippocampus on day 1 after surgery. **(G,H)** Average intensity and average polarization index boxplot of AQP4 staining centered on blood vessel in the hippocampus on day 1 after surgery. The thick lines represent means and the shaded regions represent SEM in each group. *N* = 4–8, data were analyzed with one-way ANOVA, and expressed as means ± SEM. *p < 0.05, **p < 0.01, ***p < 0.001, and ****p < 0.0001.

The polarization of AQP4 channels in astrocytes indicates a high localization of AQP4 to the perivascular endfeet, which plays a crucial role in maintaining normal glymphatic system drainage. Loss of AQP4 localization in astrocyte endfeet can lead to depolarization and subsequently impair glymphatic system drainage (Rasmussen et al., 2018; Si et al., 2024). In addition, it has been confirmed that A1 astrocytes exhibit increased susceptibility to AQP4 depolarization (Feng et al., 2023). As shown in Figures 3A–C, surgery resulted in increased expression of the astrocyte marker GFAP in the hippocampus of the LdcLNs group compared with the other three groups on day 1 after surgery. And in line with these, the expression of A1 astrocyte marker C3 was also upregulated, indicated that surgery could lead to increased reactive astrogliosis in the LdcLNs group. In terms of AQP4 channels in astrocytes, surgery further exacerbated the increased distribution area and depolarization of AQP4 in the LdcLNs group (Figures 3D–F). Then, polarization index was applied for more accurate analysis of the distribution of perivascular AQP4 centered on vasculature in each group. The depolarization distribution of AQP4 is inversely correlated with the polarization index. As shown in Figures 3G, H, surgery also further reduced the polarization index of AQP4 in the LdcLNs group. Overall, these results suggest that surgery aggravates activation of A1 astrocytes and AQP4 depolarization in middle-aged mice with preoperative brain lymphatic drainage impairment, which may be an important mechanism for the aggravation of glymphatic dysfunction caused by surgery.

### Surgery induces neuronal damage in middle-aged mice with preoperative brain lymphatic drainage impairment

**FIGURE 4:**
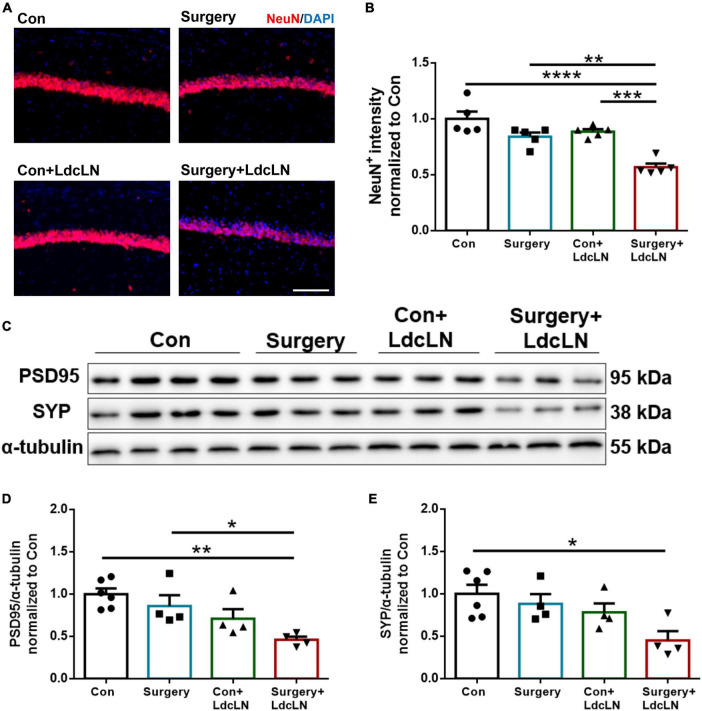
Surgery induces neuronal damage in hippocampus of middle-aged mice with preoperative brain lymphatic drainage impairment. **(A)** Representative images of NeuN immunostaining in the hippocampus on day 8 after surgery. Scale bar = 100 μm **(B)** Quantitative results of NeuN fluorescence intensity in the hippocampus on day 8 after surgery. **(C)** Representative Western blot images of postsynaptic density protein 95 (PSD95) and synaptophysin (SYP) in the hippocampus. **(D,E)** Expression of PSD95 and SYP was quantified and normalized to α-tubulin. *N* = 4–6, data were analyzed with one-way ANOVA, and expressed as means ± SEM. *p < 0.05, **p < 0.01, ***p < 0.001, and ****p < 0.0001.

In numerous animal models of neurological disorders, impaired brain lymphatic drainage can cause neuronal damage (Si et al., 2006; Wang et al., 2019; Zou et al., 2019). We hypothesize that neurons of mice with LdcLNs may also exhibit heightened vulnerability to surgical trauma. on day 7 after surgery, we observed a significant reduced expression of neurons marker NeuN in the hippocampal CA1 region of mice with LdcLNs following surgery, as compared to the other three groups (Figures 4A, B). Furthermore, in comparison to the control group, we also found that surgery resulted in a significant reduction of post-synaptic marker PSD95 and pre-synaptic marker synaptophysin levels in the hippocampus of mice with LdcLNs (Figures 4C–E). These results indicate that a synergistic effect of surgery and preoperative brain lymphatic drainage impairment on neuronal damage in middle-aged mice.

### Surgery impairs exploratory behavior and spatial working memory in middle-aged mice with preoperative brain lymphatic drainage impairment

**FIGURE 5:**
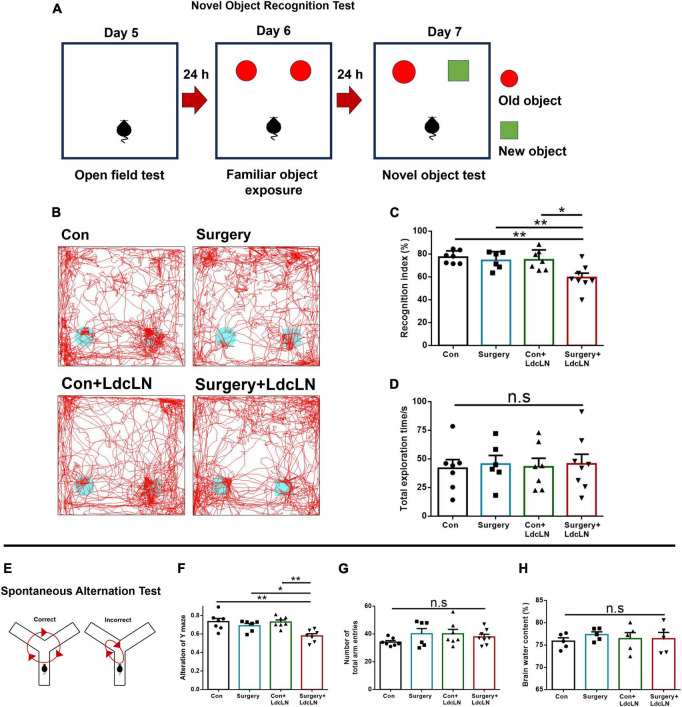
Surgery impairs exploratory behavior and spatial working memory in middle-aged mice with preoperative brain lymphatic drainage impairment. **(A)** Schematic diagram of Novel object recognition test. **(B)** Representative trace images of mice in the novel object recognition test. **(C,D)** The recognition index and total exploration time of both objects in the novel object recognition test. **(E)** Schematic diagram of the spontaneous alternation test on day 8 after surgery. **(F,G)** The correct alteration and total time of arm entries in the spontaneous alteration test of Y maze. **(H)** Brain water content in four groups on day 8 after surgery. *N* = 5–8, data were analyzed with one-way ANOVA, and expressed as means ± SEM. *p < 0.05 and **p < 0.01.

To explore the postoperative memory and learning function changes in middle-aged mice with LdcLNs, we conducted the NOR test and spontaneous alteration test of Y maze to evaluate exploratory behavior and spatial working memory on day 7–8 after surgery. In NOR test, there was no significant difference in the total exploration time among the four groups. The recognition index of LdcLNs group with surgery was significantly less than that in other three groups. Additionally, mice that underwent surgery or LdcLNs alone showed no significant difference when compared to the control group (Figures 5A–D). In the spontaneous alternation test, there was no statistically significant difference in total time of arm entries among the four groups. The correct alterations in the LdcLNs group with surgery were significantly less than that in the other three groups. Mice that underwent surgery or LdcLNs alone did not exhibit a significant difference compared to the control group (Figures 5E–G). Furthermore, our findings demonstrate no significant difference in brain water content among the four groups, thereby excluding any potential effects of brain edema that might have resulted from LdcLNs on behavioral tests (Figure 5H). In summary, our data reveal that while surgery or LdcLNs alone does not induce impaired exploratory behavior and spatial working memory in middle-aged mice, it is noteworthy that under the condition of preoperative brain lymphatic drainage impairment, surgery can cause postoperative neurocognitive disorder in middle-aged mice.

### Surgery induces neuroinflammation in middle-aged mice with preoperative brain lymphatic drainage impairment

**FIGURE 6:**
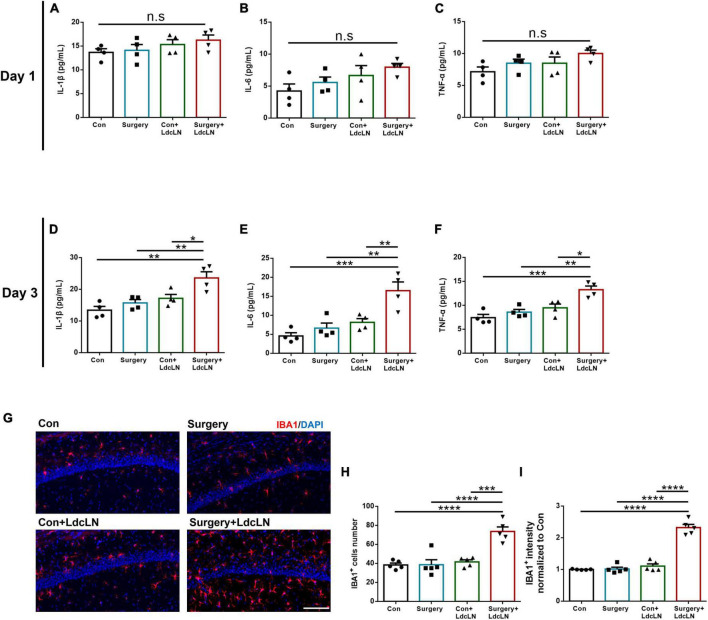
Surgery induces microglial activation and increased inflammatory cytokine secretion in hippocampus of middle-aged mice with preoperative brain lymphatic drainage impairment. **(A–C)** Levels of IL-1β, IL-6 and TNF-α in the hippocampus on day 1 after surgery. **(D–F)** Levels of IL-1β, IL-6 and TNF-α in the hippocampus on day 3 after surgery. **(G)** Representative images of IBA1 immunostaining in the hippocampus after day 3 of surgery. Scale bar = 100 μm **(H,I)** Quantitative results of IBA1 cells number and fluorescence intensity in the hippocampus on day 3 after surgery. *N* = 4–5, data were analyzed with one-way ANOVA, and expressed as means ± SEM. *p < 0.05, **p < 0.01, ***p < 0.001, and ****p < 0.0001.

The amplification of neuroinflammation induced by surgery serves as the central mechanism underlying postoperative neurocognitive disorder (Liu et al., 2023). We postulated that in the condition of preoperative impairment in brain lymphatic drainage, surgery is more likely to induce an exacerbation of neuroinflammation, ultimately resulting in neuronal damage and the development of neurocognitive disorders. We observed a tendency for increased levels of IL-1β, IL-6, and TNF-α in the hippocampus on day 1 after surgery (Figures 6A–C), with a significant elevation evident on day 3 after surgery (Figures 6D–F). Consistent with inflammatory factors elevation on day 3 after surgery, surgery resulted in a significant augmentation in both the cells number and fluorescence intensity of IBA1^+^ microglia in the hippocampal CA1 region in the LdcLNs group compared with the other three groups (Figures 6G–I).

### Surgery induces greater activation of HMGB1/TLR-4/NF-κB pathway in middle-aged mice with preoperative brain lymphatic drainage impairment

**FIGURE 7:**
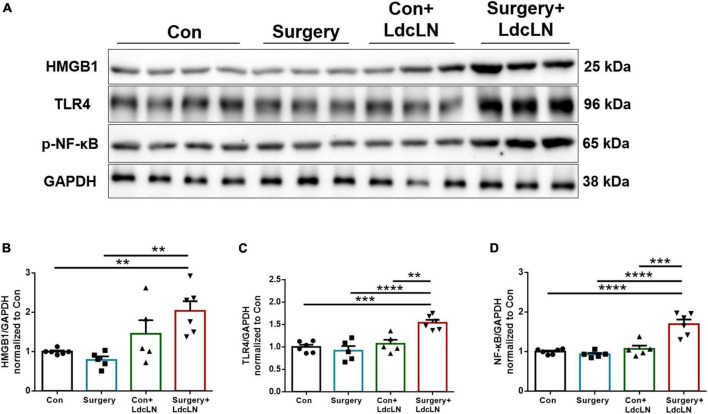
Surgery activates the HMGB1/TLR-4/NF-κB pathway in hippocampus of middle-aged mice with preoperative brain lymphatic drainage impairment. **(A)** Representative Western blot images of HMGB1, TLR-4 and NF-κB in the hippocampus on day 1 after surgery. **(B–D)** Expression of HMGB1, TLR-4 and NF-κB was quantified and normalized to GAPDH. *N* = 5–6, data were analyzed with one-way ANOVA, and expressed as means ± SEM. **p < 0.01, ***p < 0.001, and ****p < 0.0001.

It has been demonstrated that HMGB1 plays a crucial role as a DAMP in the pathogenesis of postoperative neurocognitive disorder, contributing to microglial activation through the TLR4/NF-κB signaling pathway (Terrando et al., 2016; Wang et al., 2021). On day 1 after surgery, we found an upregulation of HMGB1, TLR-4, and NF-κB expression in the hippocampus of middle-aged mice with preoperative impairment in brain lymphatic drainage following surgery (Figures 7A–D). Combined with above results, Surgery may induce neuroinflammation and glymphatic dysfunction via HMGB1/TLR-4/NF-κB pathway in middle-aged mice with preoperative brain lymphatic drainage impairment.

## Discussion

**FIGURE 8:**
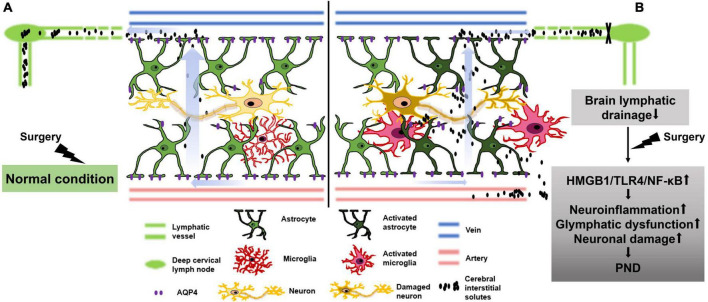
Schematic diagram illustrating that surgery exacerbates glymphatic dysfunction, neuroinflammation and neurocognitive disorder in middle-aged mice with preoperative brain lymphatic drainage impairment. **(A)** In normal condition of brain lymphatic drainage, surgical-induced elevation of cerebral interstitial solutes, such as damage-associated molecular patterns (DAMPs) and inflammatory factors etc., can be effectively eliminated through cerebrospinal fluid drainage to avoid glial cell activation, exacerbation of neuroinflammation, neuronal damage, and postoperative neurocognitive disorder (PND). **(B)** In the condition of brain lymphatic drainage impairment, surgical-induced increased DAMPs and inflammatory factors etc., can accumulate in brain parenchyma and activate the HMGB1/TLR-4/NF-κB pathway, which aggravates glial activation and the depolarization of AQP4, as well as neuroinflammation and glymphatic dysfunction. Ultimately, it results in neuronal damage and PND.

Surgery-induced neurocognitive disorders are more likely to manifest in aging and neurodegenerative populations. These populations share common characteristics of preoperative impairment in brain lymphatic drainage. In the present study, we investigated the surgery-induced neurocognitive disorder in middle aged mice with preoperative brain lymphatic drainage impairment. The results revealed that surgery can activate the HMGB1/TLR4/NF-κB pathway, thereby exacerbating postoperative neuroinflammation, glymphatic dysfunction and neuronal damage in the hippocampus, inducing neurocognitive disorder in adult mice with preoperative brain lymphatic drainage impairment. The schematic diagram of this study is shown in Figure 8.

OVA tracer can be injected into the CSF of the subarachnoid space via the cisterna magna to investigate the function of brain lymphatic drainage. Recent study has revealed the presence of an arachnoid cuff exit (ACE) point within the arachnoid barrier, facilitating fluid and molecular exchange between the subarachnoid space and the dura mater (Smyth et al., 2024). This mechanism enables the drainage of subarachnoid CSF toward the dura maters, ultimately exiting through meningeal lymphatic vessels. The present study found that both in the groups with or without surgery, LdcLNs could cause the retention of OVA-647 tracer in mLVs, denser mLVs expression, and almost no inflow of OVA-647 into the dcLNs. This suggests a significant blockage of CSF drainage from the subarachnoid space to mLVs, cervical lymphatic vessels, and dcLNs. In other words, meningeal lymphatic drainage was impaired after LdcLNs. Given that LdcLNs leads to denser LYVE1^+^ immunofluorescence staining, LdcLNs potentially cause compensatory proliferation of meningeal lymphatic vessels, which raises the possibility that the increased signal of OVA-647 may be due to the increase in mLVs.

Additionally, LdcLNs may also modulate the glymphatic system, which serves as the upstream drainage pathway for meningeal lymphatic drainage. Furthermore, we observed a reduction in both CSF inflow and outflow of the brain parenchyma in LdcLNs groups, indicating impaired glymphatic drainage function after LdcLNs. The possible reasons for the aforementioned phenomena are as follows: firstly, there is a decrease in brain lymphatic drainage ability after LdcLNs, resulting in reduced CSF flow velocity in the brain parenchyma and paravascular space; secondly, ligation of dcLNs may lead to abnormal accumulation of toxic molecules in the brain parenchyma and paravascular space, causing obstruction of the perivascular space and subsequent impairment of CSF drainage; furthermore, abnormal accumulation of toxic products in the brain parenchyma may induce damage or activation of astrocytes, leading to aberrant distribution of AQP4 polarization and resulting in glymphatic dysfunction. We observed an upregulation in GFAP expression and a downregulation in distribution of AQP4 polarization in hippocampal astrocytes following LdcLNs, consistent with glymphatic dysfunction. In the model of APP/PS1 mice and A53T mice, LdcLNs also significantly reduced CSF drainage into dcLNs and inflow into the brain parenchyma (Wang et al., 2019; Zou et al., 2019). These results suggest that LdcLNs exerts dual effects on meningeal lymphatic drainage impairment and glymphatic system dysfunction, thereby causing brain lymphatic drainage impairment in middle-aged mice.

The brain lymphatic drainage impairment can manifest in populations with aging, neurodegenerative and neurovascular diseases, as well as stroke and traumatic brain injury (TBI) (Sun et al., 2018; Chen et al., 2021; Formolo et al., 2023; Hussain et al., 2023). The impact of surgical trauma on the central nervous system of patients with brain lymphatic drainage impairment remains unclear, despite the significant number of annual surgeries required among these populations. This study found that surgical trauma aggravated the glymphatic dysfunction in middle-aged mice with preoperative brain lymphatic drainage impairment, and significantly increased the activation of A1 astrocytes and decreased AQP4 polarization. We proposed that brain lymphatic drainage impairment results in reduced clearance of surgery-induced neurotoxic molecules, such as DAMP and inflammatory factors. Consequently, this leads to a more pronounced accumulation of toxic products within the brain parenchyma, exacerbating perivascular space obstruction and triggering A1 astrocyte activation while reducing AQP4 polarization. The polarization of AQP4 plays a crucial role in driving CSF circulation in the glymphatic system. Depolarization of AQP4 can lead to a reduction in both inflow and outflow levels of brain parenchyma (Hablitz et al., 2020; Feng et al., 2023; Peng S. et al., 2023). In addition, our study found no significant increase in brain water content after LdcLNs compared with non-LdcLNs mice in both control and surgery groups, which was consistent with others research (Zou et al., 2019), suggesting that blocking brain lymphatic drainage by LdcLNs does not cause brain edema in middle-aged mice. This is likely attributed to the fact that cervical lymphatic vessels contribute to approximately 50% of CSF outflow toward cervical lymph nodes, while the remaining CSF outflow can drain from the spinal cord toward mediastinal, iliac, and sacral lymph nodes, etc (Jacob et al., 2019; Ma et al., 2019; Yoon et al., 2024). Although the ability of brain lymphatic drainage is impaired by LdcLNs for one month, CSF can compensate by flowing out through other pathways such as the spinal cord, thereby preventing the occurrence of cerebral edema.

Currently, several hypotheses exist regarding the pathogenesis of PND, encompassing central neuroinflammation, deposition of pathogenic proteins, imbalance of neurotransmitters, neuronal apoptosis, and oxidative stress; however, it is noteworthy that neuroinflammation emerges as the predominant mechanism underlying PND (Liu et al., 2023; Peng W. et al., 2023; He et al., 2024). Our results suggest that brain lymphatic drainage impairment may hamper the clearance of surgery-induced inflammatory factors, toxic substances, and oxidative stress products in the brain parenchyma. This amplifies the neuroinflammatory response, which in turn aggravates brain lymphatic drainage damage and leads to accumulation of harmful substances, resulting in neuronal damage and neurocognitive disorders. Recent studies on aged mice have shown that laparotomy can disrupt AQP4 polarization, leading to lymphatic dysfunction and PND. However, restoring AQP4 polarization can enhance glymphatic clearance of neurotoxic molecules such as IL-6 and alleviate PND (Zhu et al., 2024). Another study in a sepsis-associated encephalopathy (SAE) model also found that impairing the brain lymphatic drainage system exacerbated SAE-induced neuroinflammation and cognitive dysfunction, while promoting meningeal lymphatic drainage improved SAE (Dong et al., 2024). Therefore, brain lymphatic drainage impairment plays an important role in surgery-induced neuroinflammation and PND.

Our data also found that surgery did not cause CNS neuroinflammation, neuronal damage, or neurocognitive disorders in normal middle-aged mice. However, surgery caused microglia proliferation and activation as well as increased inflammatory factors in the hippocampus of middle-aged mice with preoperative brain lymphatic drainage impairment, leading to neuronal damage and a decline in learning and memory. Zhao et al. (2016) demonstrated surgery increases in IL-1β and IL-6 levels, along with significant microglia activation in the hippocampus of 2-month-old mice. However, no significant changes in behavior were observed in these mice (Zhao et al., 2016). Similar findings were also reported in adult mice exposed to multiple laparotomy procedures within a short period (Zhou et al., 2020). However, Wu et al. (2023) discovered that laparotomy did not induce elevated concentrations of IL-6, TNF-α and IL-1β, microglia activation, and neurocognitive disorders in 2-month-old male mice. In middle-aged mice, neither single nor multiple surgeries induced PND, despite the exacerbation of neuroinflammation observed with multiple surgeries (Lu et al., 2023; Zhang et al., 2024). These discrepancies may arise from differences in the age of mice, the extent of surgery, and the level of surgical trauma. Anyway, adult mice are less prone to neuronal damage and neurocognitive disorders following a single surgery, regardless of whether there is increased neuroinflammation after surgery or not. These findings are consistent with our findings and clinical observations. Intriguingly, Xiong’s team established a novel model for studying PND by inducing hyperhomocysteinemia in middle-aged mice through intraperitoneal injection or gene editing, based on the evidences that increased serum homocysteine levels are a common characteristic associated with aggravated neuronal damage in aging and AD (Li et al., 2024; Zhang et al., 2024). Their research enlightened us that middle-aged mice with brain lymphatic drainage impairment may serve as another new model for reducing the need for old mice and saving time and costs in studying PND. Therefore, we will next explore cognitive function changes and associated pathological changes in middle-aged mice with LdcLNs in other classic surgical models or anesthesia to compare whether this model will develop similar cognitive deficits as those in aged mice after surgery or anesthesia.

We further evaluated the HMGB1/TLR4/NF-κB pathway, which was not activated by surgery or preexisting brain lymphatic drainage impairment alone. However, under the condition of brain lymphatic drainage impairment, surgery can cause significant upregulation of the above pathway proteins. HMGB1 is a typical DAMP that can be released during various types of cell death induced by surgery trauma. HMGB1 can rapidly cross the blood-brain barrier (BBB) into the brain and its transport rates are enhanced by inflammation (Wang et al., 2021; Banks et al., 2023). The released HMGB1 binds to the receptors such as RAGE, TLR2, TLR4 and activates MyD88, leading to the activation of NF-κB signaling pathway and promoting the production and release of cytokines TNF-α, IL-6, and IL-1β (Terrando et al., 2016; Zindel and Kubes, 2020). Early studies have demonstrated a correlation between the upregulation of HMGB1 in the hippocampus and postoperative cognitive dysfunction (Li et al., 2013). Subsequently, a later study established a causal relationship between HMGB1 and PND by reporting that inhibition of HMGB1 via its neutralizing antibody effectively mitigated PND in aged rats undergoing partial hepatolobectomy (Terrando et al., 2016). Several drugs exert their pharmacological effects through the inhibition of various molecules within the HMGB1/TLR4/NF-κB pathway, resulting in reduced hippocampal inflammatory responses, suppressed microglial activation, mitigated neuronal injury and attenuated surgery-induced memory impairment (Wang et al., 2022, 2023; Yao et al., 2023). As for the glymphatic system, there are limited studies associated with this pathway. It has been reported that IL-1β can induce the expression of AQP4 through a NF-κB pathway in astrocytes (Ito et al., 2006). Other studies on Huntington’s disease (HD), a neurodegenerative disorder, NF-κB pathway can impair astrocyte function, and the treatment of intravenously injection of mesenchymal stem cells can improve the glymphatic transport via modulation of the NF-κB activity (Wu et al., 2020). However, it should be noted that the NF-κB pathway is a complex process involving multiple upstream and downstream components in various cell types. Apart from astrocytes, other resident brain or peripheral cells may also contribute to glymphatic dysfunction through the HMGB1/TLR4/NF-κB pathway. These results suggest that brain lymphatic drainage impairment may result in compromised clearance of surgery-induced HMGB1 in brain parenchyma, leading to activation of the HMGB1/TLR4/NF-κB pathway, thereby cause neuroinflammation, glymphatic dysfunction and PND.

Our studies provide new insights into aging and preoperative diseases that are susceptible to PND, suggesting that aging, AD, TBI, and other underlying neurovascular diseases or neurodegeneration may share common mechanisms of brain lymphatic drainage impairment. these conditions progress slowly without causing neuroinflammation or cognitive impairment, while surgery exacerbates the drainage impairment beyond their compensatory capacity, leading to PND or even long-term dementia. The brain lymphatic drainage system may be a critical target for PND administration. Surgery often accompanies with inflammation, sleep disturbances, and anesthesia etc., all of which can impede brain lymphatic drainage (Ren et al., 2021). Optimizing perioperative management to mitigate related factors that hinder brain lymphatic drainage may effectively reduce the morbidity of PND. This study has some limitations. Firstly, we only explored surgery-induced neurocognitive disorder in middle-aged mice with perioperative brain lymphatic drainage impairment; further studies will be conducted in young adult mice. Secondly, we found that surgery induces the activation of HMGB1/TLR-4/NF-κB pathway in preoperative brain lymphatic drainage. However, it remains unclear how different types of CNS cells participate in neuroinflammation, glymphatic dysfunction, and PND through the HMGB1/TLR-4/NF-κB pathway. Moving forward, we will elucidate the specific cellular mechanisms underlying surgery-induced neuroinflammation and glymphatic dysfunction in mice with perioperative brain lymphatic drainage impairment.

In conclusion, our data demonstrate that surgery activates the HMGB1/TLR4/NF-κB pathway, exacerbates glymphatic dysfunction and neuroinflammation, ultimately results in neurocognitive disorder in middle-aged mice with preoperative brain lymphatic drainage impairment. These results suggest that brain lymphatic drainage impairment may be one of the underlying mechanisms related to the progression of PND, and restoring its function may serve as a potential strategy against PND.

## Data availability statement

The original contributions presented in this study are included in this article/supplementary material, further inquiries can be directed to the corresponding authors.

## Ethics statement

The animal study was approved by the Animal Ethical and Welfare Committee of Sun Yat-sen University. The study was conducted in accordance with the local legislation and institutional requirements.

## Author contributions

XZ: Conceptualization, Data curation, Formal analysis, Investigation, Methodology, Project administration, Software, Writing – original draft, Writing – review & editing. JL: Conceptualization, Data curation, Formal analysis, Investigation, Methodology, Project administration, Software, Writing – original draft, Writing – review & editing. PY: Conceptualization, Data curation, Formal analysis, Investigation, Methodology, Project administration, Software, Writing – original draft, Writing – review & editing. SW: Data curation, Methodology, Software, Writing – original draft. HL: Data curation, Methodology, Software, Writing – original draft. WH: Data curation, Methodology, Software, Writing – original draft. DL: Conceptualization, Data curation, Formal analysis, Funding acquisition, Investigation, Methodology, Project administration, Software, Supervision, Writing – original draft, Writing – review & editing. MC: Conceptualization, Data curation, Formal analysis, Funding acquisition, Investigation, Methodology, Project administration, Software, Supervision, Writing – original draft, Writing – review & editing.
